# Supplementary material for: Preparation, Characterization, and Anti-Cancer Activity of Nanostructured Lipid Carriers Containing Imatinib
Source: Pharmaceutics. 2021 Jul 16;13(7):1086. doi: 10.3390/pharmaceutics13071086 (PMC8309103; doi:10.3390/pharmaceutics13071086)
Supplement: Supplementary file 1 [file pharmaceutics-13-01086-s001.zip › pharmaceutics-1272791-supplementary.pdf]

pharmaceutics

MDPI

Supplementary Materials: Preparation, Characterization, and Anti-Cancer Activity of Nanostructured Lipid Carriers Containing Imatinib

Hafiz A. Makeen, Syam Mohan, Mohamed Ahmed Al-Kasim, Muhammad Hadi Sultan, Ahmed A Albarraq, Rayan A. Ahmed, Hassan A. Alhazmi and Mohammad Intakhab Alam

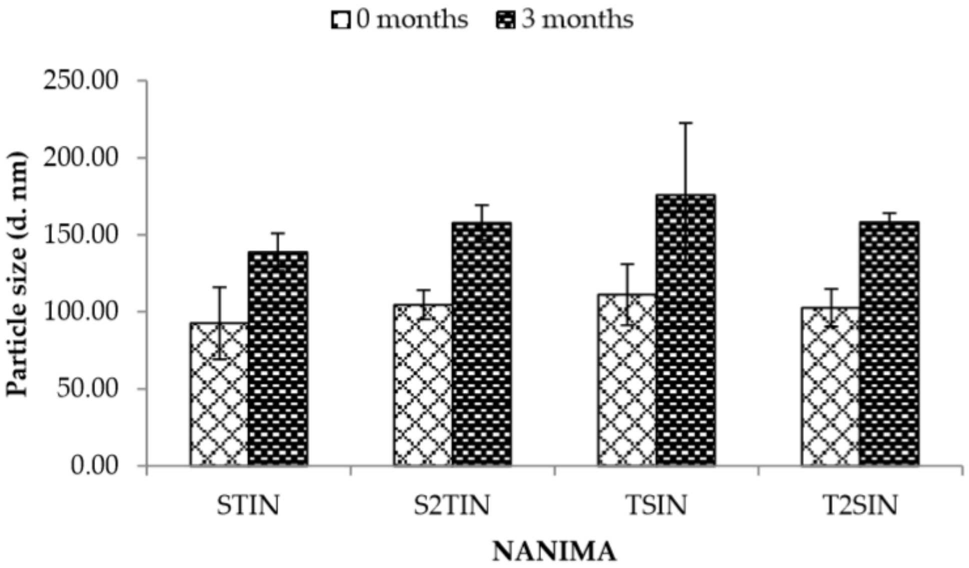

Figure S1. Effect of storage on the particle size of NANIMAs after three months of storage. The increase in particle size was observed to be nonsignificant in all NANIMA formulations.

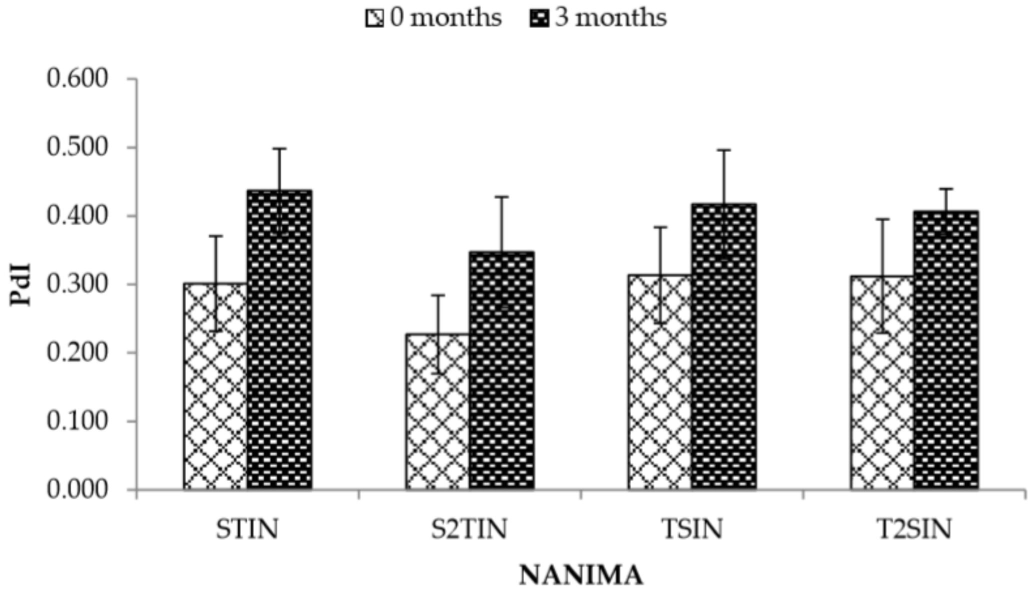

Figure S2. Effect of storage on the polydispersity index (PdI) of the NANIMAs after three months of storage. STIN exhibited the highest increase ($p = 0.065$) and S2TIN exhibited the lowest increase among all of the NANIMAs ($p < 0.05$).

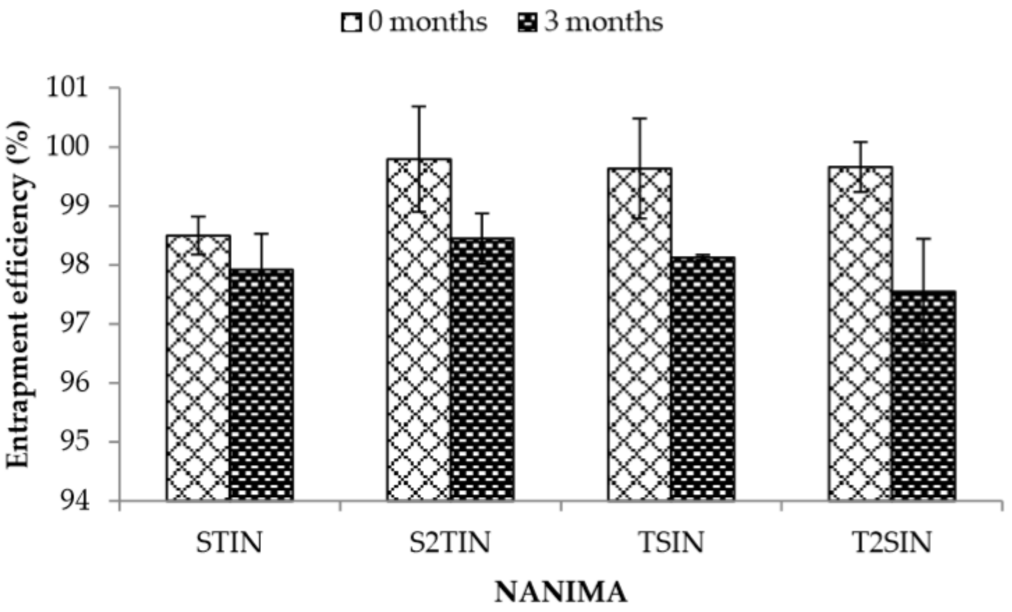

Figure S3. Effect of storage on the leakage of IMA from the NANIMAs after three months of storage. All of the NANIMAs were assessed to show a decrease in EE, including T2SIN ($p < 0.05$), TSIN ($p < 0.05$), S2TIN ($p < 0.05$), and STIN ($p > 0.05$).

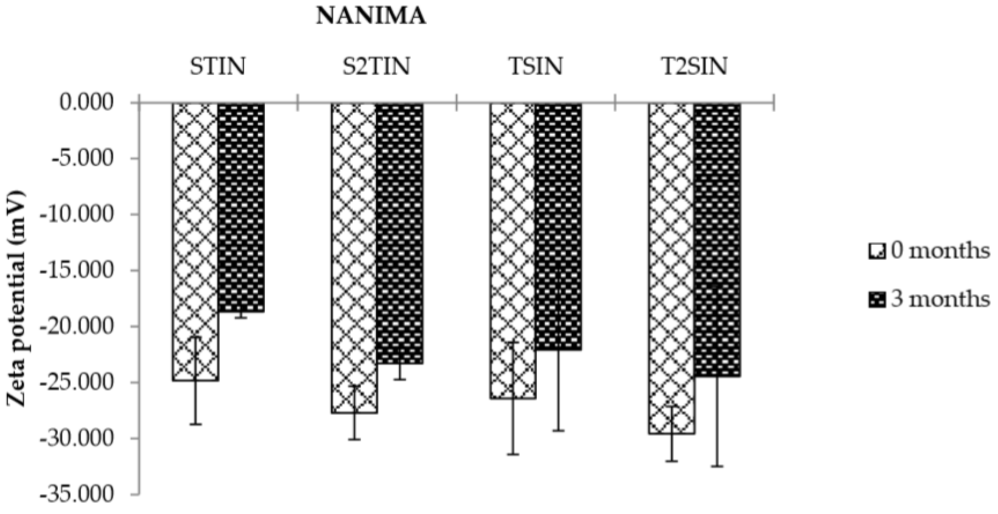

Figure S4. Effect of storage on the zeta potential. All of the NANIMAs were found to exhibit a nonsignificant ($p > 0.05$) decrease in ZP. However, STIN exhibited a significant reduction in ZP ($p <$ 0.05).
